# Views on deceased organ donation in the Netherlands: A q-methodology study

**DOI:** 10.1371/journal.pone.0216479

**Published:** 2019-05-24

**Authors:** Daphne Truijens, Job van Exel

**Affiliations:** 1 Erasmus University Rotterdam, Institute for Philosophy & Economics (EIPE), Rotterdam, the Netherlands; 2 Erasmus University Rotterdam, Erasmus School of Economics, Rotterdam, the Netherlands; 3 Erasmus University Rotterdam, Erasmus School of Economics, Rotterdam, the Netherlands; 4 Erasmus University Rotterdam, Erasmus School of Health Policy & Management, Rotterdam, the Netherlands; Fordham University, UNITED STATES

## Abstract

In many countries, such as the US, Germany, France, and the Netherlands, governments are dealing with a great shortage of organ donors. Even though people generally show positive attitudes towards organ donation, they often do not actually register as organ donors themselves. This study’s objective was to explore prevailing viewpoints among the Dutch population on deceased organ donation and the relation between aspects of the viewpoints potentially influencing the decision to register as an organ donor. Although substantive research about attitudes on organ donation has been conducted, this is the first study investigating people’s viewpoints focusing on the *relation* between beliefs, tastes, preferences, motives, goals and other constituents underlying people’s viewpoints on organ donation, such as the role of the media and public policies. This Q-methodology study revealed four viewpoints: “not donating your organs is a waste”, “it does not go with my religion”, “my family should decide”; and “it’s a good deed, but I’m doubtful”. These viewpoints convey information on potential reasons for the gap between people’s favourable attitudes towards organ donation and the low number of actual registrations, and opportunities for policy makers to address certain target groups more adequately.

## Introduction

In many countries, such as the US, Germany, France, and the Netherlands, governments are dealing with a great shortage of organ donors (e.g., [1–4]). Even though people generally show positive attitudes towards organ donation, they often do not actually register as organ donors themselves [5–12]. In order to create greater awareness of organ donor scarcity and ultimately recruit more potential organ donors, public policy makers have taken up a wide range of actions, including the development of extensive media campaigns (e.g., [13–15]). In this context, public policy increasingly relies on behavioural insights from economics and psychology, such as research which shows that people tend to stick with default positions and are therefore more likely to become an organ donor under an opt-out system (e.g., [16–23]). For instance, in the Netherlands recently a law passed which will change the organ donation registration system from an opt-in to an opt-out system. Positive effects of these type of behavioural public policies are often explained by weakness of will or by people’s alleged indifference about the choice at issue [17, 24]. Yet, these explanations are considered controversial (e.g., [24]), and the question of how people think about organ donation and the choice to register as organ donors, including the role of default positions, is still a matter of debate (e.g., [25]).

In order to develop adequate and effective public policies to promote organ donor registration, it is essential to gain insight in people’s viewpoints on organ donation. Understanding the existing viewpoints on this issue is important to be able to identify target groups for policy, and how to approach them adequately and effectively. Some citizens will have made up their mind about registering as an organ donor, others might still be in doubt, for a variety of reasons. These various groups require different approaches in terms of raising awareness and knowledge, and incentivizing registration. Moreover, it may inform about potential drawbacks of policies for particular subgroups that need consideration. For instance, implementing an opt-out policy might disadvantage people who are against organ donation but for whom information on the registration process and instructions for opting out are less accessible. In this context, several studies have investigated people’s attitudes towards organ donation and their willingness to register as an organ donor (e.g., [26–28]). Furthermore, substantive research has been done on sociodemographic differences in attitudes, knowledge and behaviour towards organ donation (e.g., [29–35]). However, little research has been conducted into how people’s beliefs, preferences, goals, and other elements such as the perceived role of the media and public policy are related and together constitute different viewpoints on organ donation that exist among the public. As a consequence, there is insufficient knowledge of what characterises and distinguishes people’s viewpoints, and how this could inform the development of adequate and effective policies to promote organ donation. The aim of this research was to contribute to the literature by exploring more in detail the prevailing viewpoints about deceased organ donation among the public in the Netherlands, and their implications for the decision to register as an organ donor. Deceased organ donation refers to organ donation after death, thereby excluding “living organ donation” of, for instance, kidneys.

## Methods

### 2.1 Q-methodology

We used Q-methodology to explore the viewpoints about organ donation among the public in the Netherlands. Q-methodology combines qualitative and quantitative methods to discover what constitutes the view of individual respondents about a value-laden topic, identify shared viewpoints among respondents, and the consensus and differences between these viewpoints [36, 37]. In this context, viewpoints are defined as an attitude how one thinks of organ donation, constituted by a coherent set of individual beliefs, preferences and goals, the perceived role of the media and public policy, and other elements potentially influencing how one perceives the decision to register as an organ donor (for more detailed discussions of the definition of viewpoints in the context of Q-methodology studies (e.g., [38–40]). Because it commonly relies on purposive sampling, Q-methodology is useful to explore the variety of viewpoints that exists within a population but does not provide information about the proportion of the population that holds a particular viewpoint [41]. In a Q study, respondents are asked to rank a comprehensive set of statements concerning the topic at issue and to explain their ranking of the statements in a follow-up interview. The shared viewpoints are identified using by-person factor analysis, which searches for correlations between respondents’ answers across a sample of variables [42]. The underlying assumptions are that within a certain population only a limited number of viewpoints exist, thereby paving the way for an analysis of types of viewpoints rather than assessing each individual’s viewpoint by itself, and that respondents who rank the statements in a similar way share a similar viewpoint on the topic [43]. In this study we apply common techniques in Q-methodology for the design of the study, and collection and analysis of the data. Readers interested in more detail about the background of the method and common/ best practices in applications of the method are referred to Watts and Stenner [37] or McKeown and Thomas [44]. Q-methodology has been applied before in the domains of health promotion, education and behaviour (e.g., [38, 45–50]).

### 2.2 Development of the statement set

For this study, a set of 43 statements was constructed, containing a large variety of aspects relating to the decision to register for deceased organ donation. In order to enable all respondents to express their view it is important that the statement set is comprehensive and balanced, broadly representative of the issues of debate on this topic in the literature and among the target population For this purpose, we first identified potentially relevant aspects to cover from common theoretical models about health intentions and behaviour like the Health Belief Model [51] and the Theory of Planned Behaviour [52] This concerned perceived severity, susceptibility, barriers and benefits, self-efficacy / perceived behavioural control, cues to action and subjective norms. In addition, based on the recent literature on determinants of the decision to register as an organ donor (e.g., [15, 53], a “moral beliefs” component was added.

It should be stressed that the aim of using these theoretical models was to make sure that the statement set is comprehensive and captures all relevant aspects potentially relevant for people’s viewpoints on organ donation, not to function as a model by which the causal relations of these aspects are explained.

Next, 350 statements on organ donation were extracted from academic literature, newspaper articles, radio and television documentaries, discussions on social media, and related information websites. Academic literature published between 2003 and 2017 was searched through Google Scholar and PubMed using the keywords “organ donation”, “viewpoints on organ donation”, “attitudes towards organ donation”, “opinions on organ donation”, “organ donation default positions”, “organ donation opt-in opt-out systems”. A total of 350 statements was retrieved, which were structured according to the components of the theoretical models to ensure these were all covered. Redundant, unclear and irrelevant statements were deleted, leaving an initial set of 47 statements for pilot-testing.

**Fig 1:**
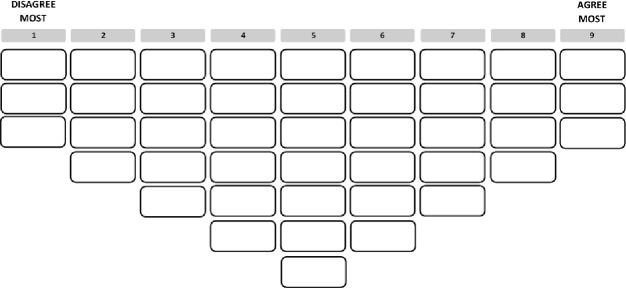
Sorting grid.

In order to check whether the statement set was comprehensive and intelligible, all other research materials were clear, and the whole approach feasible and sufficiently appealing to participants, two pilot studies were conducted. Respondents varying in age, gender, education level, ethnicity and religiosity were presented with the draft research material and asked to critically review the information letter, instructions and statement set. Based on a first pilot study in which five respondents took part, several changes were made to the statement set, including the rephrasing of several statements, deleting three statements that were considered irrelevant by the respondents and combining two statements that were regarded as too similar. Next, another five respondents and two researchers experienced with Q-methodology were asked to review the adjusted study materials. As a result, one more statement was deleted, four statements were rephrased and one statement was added, resulting in a set of 43 statements for the main study. Moreover, because participants in both rounds of the pilot indicated that the sorting grid did not provide sufficient opportunity to rank statements in the least and most agree categories, the number of spaces for placing statements in the two outer columns of the grid was expanded from two to three (Fig 1).

### 2.3 Data collection

In the main study, a total of 30 respondents were interviewed (Table 1). Provided they are well-selected, this is generally considered an adequate sample size for exploring the views on a topic [37]. Respondents were recruited via an external agency and sampled purposively to represent demographic and cultural diversity. Based on the literature, the characteristics gender, age, education level and religion were used for sampling.

**Table 1:** Characteristics of the sample.

Characteristic	%
Gender	
Male	16
Female	14
Age	
18–35	11
35–70	19
Highest finished education	
Lower education (primary school, high school, vocational training)	18
Higher education (BA or MA / applied science or university level)	12
Religion & Philosophy of Life	
Atheism	9
Christianity	7
Judaism	3
Islam	6
Buddhism	3
Hinduism	2

At the start of the interview, respondents received an information letter informing them about the aim of the study, the content of the interview, and the processing and analysis of the data. Next, respondents received an informed consent form and were instructed that they could stop the interview at any time, for any reason. Respondents were required to provide informed consent and to finish the interview to be included in the study.

After the consent form was signed, the respondent received the set of 43 statements printed on cards in a random order. In order to ensure a correct understanding of the statements, the researcher would first read each statement out aloud after which the respondent would read the card herself, briefly comment and then place the card on one of three piles, for statements they agreed with, disagreed with, or felt neutral about. After all statements were placed on one of the three piles, respondents were instructed to read the statements they had placed in the ‘agree with’ pile once again and to rank them according to the level of agreement on the right side of the sorting grid (Fig 1). After that, they ranked the statements they had placed in the ‘disagreed with’ pile on the left side of the grid, and those they had placed in the ‘neutral’ pile in the spaces left in the middle of the grid.

After completing the ranking exercise, respondents were asked to explain the placement of certain statements on the sorting grid; all respondents elaborated on the statements placed in the two outer columns of the grid, and depending on time, on randomly chosen other statements. The interview ended with a short questionnaire consisting of closed questions about socio-demographic characteristics and whether they had registered their preference for organ donation or intended to do so.

The interviews took approximately 45 minutes and were conducted on Erasmus University campus. Respondents received 25 Euros in cash as financial compensation for their time. The study protocol was approved by the Medical Ethics Review Board of the Erasmus Medical Centre (reference number MEC-2017-221).

### 2.4. Data analysis

The 30 rankings of statements were analysed using by-person factor analysis (i.e., centroid factor extraction followed by varimax rotation) in the dedicated software package PQMethod [54]. These common techniques for Q-methodology [37] identify groups of respondents that have ranked the 43 statements in a similar way. Inspection of statistical information (i.e., explained variance and number of defining variables per factor) and the qualitative materials collected during the interviews resulted in the choice for a four-factor solution. For each of these factors a composite ranking of the statements was computed based on the rankings significantly associated with the factor and their factor loadings as weight. Such composite ranking represents how a person perfectly correlated with the factor would have ranked the 34 statements. The four factors were first interpreted based on this composite ranking of the statements per factor, considering in particular the characterising statements (i.e., those ranked in the two outer columns at each side of the composite ranking) and the distinguishing statements (i.e., those ranked statistically significantly different in a factor as compared to all other factors) of each factor. Subsequently, the qualitative materials from the interviews of respondents associated significantly with a factor (p < .05) were used to refine the check and interpretation of the factors. A number of citations from respondents were used to illustrate the interpretations.

## Results

The final sample consisted of 30 respondents. Table 1 shows the characteristics of the sample. The four factors explained 47% of the variance in the rankings of statements, and 28 respondents were significantly associated with one of the factors (p < .05). Factor correlations ranged from 0.15 for factors 3 and 4 to 0.48 for factors 1 and 2. Table 2 presents the composite rankings of the statements for each of the four factors.

**Table 2:** Factor scores.

Statement	Viewpoint			
	Not donating my organs would be a waste	It does not go with my religion	My family should decide	It is a good deed, but I‘m doubtful
1. 1. I might need a donor organ myself one day	+3	+3	0	+2
2. 2. I expect that, if really necessary, there always will be a donor organ available	-1	0	-1	-1
3. 3. I expect that people waiting for a donor organ may contract health damage	+3	+2	+4	+3
4. 4. People in need of a donor organ are not able to function normally	0	+2	+1	+2
5. 5. Information in the media helps me to determine my preference for registering as an organ donor	+1	0	0	0
6. 6. By donating my organs I can pass on the gift of life to someone else	+4	+4	+3	+1
7. 7. By donating my organs I add meaning to my life	0	-1	-1	+4*
8. 8. By donating my organs I can reduce the suffering of the recipients	+4	+1	+3	+3
9. 9. I like the idea that if I donate my organs part of me will live on after I die	-1	0	-2	0
10. 10. Donating my organs makes me proud	0	+1	+1	+3
11. 11. I think it would be a waste not to donate my organs	+4*	-1	0	+1
12. 12. I would mind donating organs that are visible to my loved ones at my farewell	0*	+3	+2	+2
13. 13. I need my organs for the afterlife	-3	-4	-2	-3
14. 14. My family would not have any problems with me donating my organs	+2*	-3	0	-1
15. 15. I am afraid that when I have registered as an organ donor, doctors may remove my organs while I am still alive	-3	+1*	-3	-4
16. 16. I am afraid that when I have registered as an organ donor, doctors my stop my treatment earlier	-2	-1	-1	-1
17. 17. I am afraid that if I donate my organs, they will end up on the black market	-4	-4	-3	+2*
18. 18. I dislike the idea that if I donate my organs, others will see my naked body	-4	-1*	-4	+1*
19. 19. I would donate some of my organs, but not all	-2*	+1	+1	+1
20. 20. I talk with my friends about organ donation	0	+2	-2*	-4*
21. 21. I find the choice whether to donate my organs difficult	-2	0	+3*	-1
22. 22. Registering your preference for organ donation takes a lot of time and energy	-2	0*	-3	-3
23. 23. I prefer not to think about organ donation	-1	-3	0*	-4
24. 24. I’m afraid that if I donate my organs, someone is kept alive who does not live a good life	-2*	0	-1	0
25. 25. There is sufficient information available about organ donation	+3	-2*	+1	+1
26. 26. I have sufficient knowledge about organ donation	+1*	-3	0	-2
27. 27. I know how to register and unregister as organ donor	+2*	-4	+3*	-1
28. 28. Whenever I’m sure about my preference for donating my organs, I will register it	+3	-3*	+2	+3
29. 29. Organ donation should be compensated financially	-3	-2	-1	-2
30. 30. The family should have the last word about organ donation	-4	-1	+4	+4
31. 31. People who have registered as organ donors should be given priority when they need a donor organ themselves	+1	+1	+1	-3*
32. 32. By donating your organs you do a good deed	+2	+4	+4	+4
33. 33. If you would like to receive a donor organ might you need one, you should also be willing to donate your organs	+1	+3	+2	-2*
34. 34. If you are not registered as organ donor, you should also not be eligible for a donor organ yourself	-1	+1	-4	-3
35. 35. Organ donation is a moral duty	+1	-2	-2	+1
36. 36. Organ donation does not go with my religious beliefs	0	+4*	-4*	0
37. 37. Removing organs from people who are brain dead but are kept alive by machines, is murder	-1	+3	+1	-2
38. 38. One should leave the dead in peace	-3	0	-3	-2
39. 39. The government should not interfere with organ donation, it is an individual matter	0	+2	+2	0
40. 40. People should receive a reward as a nudge to register as organ donor	-1	-2	0	0
41. 41. My loved ones find it important that I register as organ donor	+1	-2	-2	0
42. 42. I talk with my family about organ donation	+2*	-1	-1	-1
43. 43. People who have not registered their preference for organ donation should receive a reminder	+2	+2	+2	+2

### Viewpoint 1 –Not donating my organs would be a waste

This can be considered a pro organ donation perspective, as in this viewpoint the perceived benefits of becoming an organ donor clearly outweigh the perceived barriers. People with this view sympathize with the suffering of people waiting for a donor organ (statement 3 receives factor score +3: st.8, +3), realize they might need an organ someday themselves (st.1, +3) and emphasize benefits of organ donation such as diminishing the suffering of other people (st.8, +4) and being able to pass on life to others (st.6, +4). Distinguishing for this view is the notion that not donating your organs is considered a waste (st.11, +4) as they are of no use to yourself after death (st.13, -3; st.38, -3): “*I really don’t see why you would choose not to donate your organs*. *If you cannot use your organs anymore yourself*, *why wouldn’t you help someone else*?” In line with this, no distinction is made between which organs to donate or not (st.19, -2): “*you no longer need any of them yourself*, *so as far as I am concerned they can take them all*”; or to whom (st.24, -2).

People with this view see themselves as efficacious. Finding the relevant information is considered as relatively easy (st.22, -2) and they feel they have sufficient knowledge about organ donation (st.25, +1; st.26, +3) and about how to register or unregister as organ donor (st.27, +2): “*Sure*, *it takes some time*, *but you search on google*, *fill out a short form and there you go*”. They discussed the matter with their family (st.42, +2), who do not object to them being organ donor (st.14, +2). More than in the other views, they agreed that their family members encouraged them to register as an organ donor (st.41, +1). Still, they strongly disagreed that family members should have the last say on whether their organs eventually will be donated (st.30, -4), even if family members would object to the donation.

Trust in the healthcare system is widespread within this group. Respondents expected that medical professionals will treat their dead body and its organs with respect (st.15, -3, st.16, -2), and the possibility that the donated organs end up on the black market is considered negligible (st.17, -4). All in all, the choice to register as organ donor was not difficult for them (st.21, -2). People with this view either already had registered as organ donor or stated their intention to do so (st.28, +3), and do not see the need for incentivizing the decision to register (st.29, -3; st.40, -1).

Based on this description, we labelled this viewpoint “Not donating my organs would be a waste”. Thirteen respondents were statistically significantly associated with this viewpoint.

### Viewpoint 2 –It does not go with my religion

People with this view strongly agree that organ donation does not match their religious beliefs (st.36, +4). This is not because they expect they will need their organs in the afterlife (st.13, -4), as they believe body and soul will be separated after death. But the body is borrowed and should be returned, and thus be preserved. At the same time, they strongly agreed with the statement “By donating your organs you do a good deed” (st.32, +4) and often even presented a religious reason for this. According to them, people receive the gift of life from God and by organ donation you could pass on this gift to someone else (st.6, +4). Moreover, one day you might need an organ yourself (st.1, +3), and you should be willing to give if you wish to receive (st.33, +3). People holding this view therefore see organ donation as a “paradox” or “personal challenge” that concerns them (st.23, -3); as one of the respondents explained: “*Giving in general is considered a good deed and I think it is very important to give*. *So*, *for myself*, *I would really like to become an organ donor*. *But my religion forbids me to become an organ donor*, *because you should consider your body to be a loan from God and return it to Him*. *This is difficult for me*, *because I think giving and helping other people is an import part of my religion and of who I am*, *but I have to respect God’s will*, *even though I do not understand it*”.

Organ donation it is not a common topic of family conversion (st.42, -1). Family members are said to hold a similar religious perspective on this topic and would therefore not support them registering as organ donor (st.14, -3; st.41, -2). However, while they value the opinion of their family highly, the family should not have the last say in the decision to donate their organs (st.30, -1); it is considered to be an individual matter, for which one is personally accountable when faced with God. Notwithstanding, more than those with other views, this group does discuss the topic of organ donation with friends (st.20, +2).

People defining this view say they view would not officially register their preference for becoming organ donor or not (st.28, -3) because under the current opt-in system the only reason to register would be to become organ donor; and that’s not what they want, after all. In line with this, they were neutral regarding how difficult it is to register as an organ donor (st.21, 0). They have little knowledge and information about the registration procedure for organ donation or the transplantation process (st.26, -3; st.25,-2), and stated not to know how to register (st.27, -4); the whole procedure is something “*that you just don’t really think about*”.

Based on this description, we labelled viewpoint two “It does not go with my religion”. Two respondents were statistically significantly associated with this factor.

### Viewpoint 3 –My family should decide

People with this view consider the choice to register as an organ donor as very difficult (st.21, +3), which can best be characterized by a dilemma. On the one hand, they see various benefits of organ donation. For instance, they strongly agreed with the statement that organ donation is a good deed (st.32,+4) that would prevent further suffering or perhaps even cure people with severe health problems (st.3, +4; st.6, +3; st.8, +3). On the other hand, respondents argue that the potential suffering of family members constitutes a considerable barrier for them to donate their organs, even though they are not sure whether their family members would actually have problems with them becoming an organ donor (st.42, -1; st.41. -2; st.14, 0). People defining this view often emphasized the rapidity of transplantation and expressed a fear of adding extra trauma to family members if their dead body would be taken away so shortly after death. In addition, respondents viewed the incompleteness of the body after the transplantation as difficult for family members, especially in cases when this would be visible (ref 12, +2). One respondent explained: “*Personally*, *I find the decision to become an organ donor very difficult*. *My children are the main reason*. *I think the moment to say goodbye must be very difficult and terrible for them*, *especially if you know your mother’s body is missing parts*. *The medical staff also has to take the body away within 24 hours after death in order for the transplantation to be able to succeed*. *I think this time constraint is especially difficult for the family of the deceased*, *not being able to stay with the deceased so soon after she passed away must be very unpleasant*.*”* Since according to this group, the impact of organ donation is on the family of the deceased rather than on the deceased herself, they strongly agreed with the statement that family members should have the final say in deciding whether the organs of the deceased will be donated (st.30, +4). As this suggests the decision to donate is made post mortem by their family, not being registered as organ donor should not have consequences for their eligibility to receive a donor organ, if needed (st.34, -4).

People with this view claim to know well how to register or unregister as organ donor (st.27, +3), do not think it is too much of an effort (st.22, -3) or conflicts with their philosophy of life (st.36, -4), and do not see significant problems with donating (st.15, -3; st.16, -1; st.17, -3; st.18, -4; st.38, -3). However, they are quite doubtful about whether they have sufficient knowledge on organ donation (st.26, 0) or sufficient information is available to them (st.25, 1).

Based on this description, we labelled viewpoint three “My family should decide”. A total of 8 respondents were statistically significantly associated with this factor.

### Viewpoint 4—It is a good deed, but I‘m doubtful

Whereas respondents holding the other viewpoints repeatedly stated that “*organ donation is something that you do for others*, *not for yourself*, *to give yourself a good feeling*”, these people see donating your organs as a good deed (st.32, +4) and they would feel proud of themselves (st.10, +3) for doing something that could impact another person’s life positively (st.8, +3; st.3, +3). In this way, registering as organ donor would give more meaning to their own lives (st.7, +4). As one of the respondents defining this view explained: “*registering as an organ donor shows that you value life*, *not only your own but also the life of others*”. They argued that by registering as organ donor one would endorse the conviction that life is worth living.

Even though people with this view seem to have a positive attitude towards organ donation, they also put forward various barriers to registering as organ donor that are primarily related to fears and unease about the donation process. Distinguishing for this viewpoint were the fear that donated organs might be sold on the black market (st.17, +2), as well as unease with the possible visibility of removed organs for family members (st.12, +2) and their naked body being exposed to others during the process of transplantation (st.18, +1). Also distinguishing for this viewpoint is the disagreement with the statements saying that if you want to receive a donor organ might you need it, you should be willing to register as donor yourself (st.33, -2) and that priority should be given to registered donors in case they need an organ (st.31, -3). They also disagreed that those who have not registered as donor should not be eligible for a donor organ themselves (st.34, -3). They explained these conditions are too strong; there could be many valid reasons not to become an organ donor, such as the opinion of the family or religious reasons, and severe suffering or even death would be too serious a consequence.

People with this viewpoint doubt about the decision to register as organ donor. It is not a subject they avoid (st.23, -4), but also not one they talk about with friends (st.20, -4) or family (st.42, -1). They are generally not very well informed about the organ donation process (st.26, -2) or about how to register as organ donor (st.27, -1), and not quite so sure what information is available (st.25, +1). They think registering would not require much effort (st.22, -3) and intend to do it once they’ve made up their mind (st.28, +3), but at the same time feel that their family should eventually have the final say (st.30, +4).

Based on this description, we labelled viewpoint four “*It is a good deed*, *but I‘m doubtful*”. A total of 5 respondents were statistically significantly associated with this factor.

## Discussion

This aim of this study was to explore the prevailing viewpoints on deceased organ donation among the public in the Netherlands, and their implications for the decision to register as an organ donor. Attitudes about organ donation have been studied extensively before using survey research, but this study contributes by exploring the diversity in relations between the broad range of underlying beliefs and other constituents of people’s views on organ donation. Using Q-methodology, we found four prevailing viewpoints: “not donating your organs is a waste”, “it does not go with my religion”, “my family should decide” and “it’s a good deed, but I‘m doubtful”.

The first two viewpoints are most distinct from each other, representing positions in favour and against registering as an organ donor. For people with the first viewpoint (“not donating your organs is a waste”) the potential benefits outweigh the potential barriers. The perception of self-efficacy was also relatively high in this group, with respondents indicating that they had sufficient information and knowledge about organ donation and claiming that “*as soon as I knew I wanted to become an organ donor*, *I signed up*”. Explanations regarding the placement of statements on the ranking grid collected during the interview revealed that for this group conversations with friends and family and encouragement by the family were important cues to action, and to a lesser extent media attention for organ donation. Answers to follow-up questions revealed that the majority of respondents who defined this viewpoint already had registered as organ donor, and otherwise stated their intention to do so in the near future.

In viewpoint two (“it does not go with my religion”), on the other hand, potential barriers clearly outweigh potential benefits, self-efficacy is low and there are few cues to action. Respondents who hold this perspective generally had little knowledge and information about organ donation. Arguably, they do not experience the need to look for such information, considering the fact they had already “*made up their mind*”. However, the qualitative materials of the interviews show that these respondents were generally dissatisfied with the type of information one can find in the media. Commercials and other information are considered superficial and understood as promotion material for registering as organ donor rather than an objective overview of different arguments and viewpoints on organ donation. As one respondent stated: “*The information I see only tells you to donate your organs because it can help other people*. *But it doesn’t really tell you anything about the up- and downsides of organ donation*. *I think the government should show examples that really resonate with all sorts of citizens*. *They should talk about the procedure of organ donation*, *but also discuss various spiritual and religious points of view”*.

Viewpoints three and four showed no clear overweight of benefits or barriers. In viewpoint three (“my family should decide”), the perceived barriers were centred on the potential harm for family members rather than for themselves. These respondents often had not registered their preference for organ donation. Not because they are indifferent, too lazy or forgot, but because they do not have a clear preference on this complicated matter. Those who had registered or stated they intended to do so in the near future, still wanted their family to have the last word: “*I would like to become an organ donor*, *under the condition that my family does not object*”.

Respondents with viewpoint four (“it’s a good deed, but I‘m doubtful”) were generally positive towards organ donation, but were not well informed and expressed several fears regarding the donation process. For instance, a fear of their organs ending up on the black market and an uneasy feeling about others seeing their naked body when their organs would be removed. Like those with viewpoint three, they see an important role for the family; not because of the potential trauma for family members, as in viewpoint three, but because they are uninformed and undecided. As one respondent defining this view stated: “*I should do it because it helps other people*, *but it does make me feel a bit uneasy*. *I don’t really have a preference because of these pros and cons*, *so my family should decide*. *That is less difficult for them than for me*, *I think*, *although it still may be aggravating*.”

A striking finding of this study is that people with all views appear to appreciate the benefits of organ donation, and therefore have a positive attitude towards the topic. This is quite obvious for people with the “not donating your organs is a waste” view. Those with the other three views also all consider organ donation as a good deed, but those with the “it does not go with my religion” view will not register because of their philosophy of life, those with the “my family should decide” view hand the decision to their loved ones, whereas those with the “it’s a good deed, but I‘m doubtful” view appear not to be sufficiently involved with the topic to form a preference and come to a decision.

Another striking finding was the consensus among participants in the study about the neutral ranking of the statement: “information in the media helps me to determine my preference whether to become an organ donor”. Respondents explained that current information campaigns “*didn’t really help*, *but maybe it starts a conversation every now and then*”. Some argued that information in the media only stresses the relief in suffering for others, but does not provide any insight in the transplantation process, the communication with the family, and potential disadvantages of organ donation. Many respondents suggested it would be helpful to have a decision aid, something resembling the quizzes one can take during election times to learn which political party best represents your principles and interests. It should be noted that the interviews were conducted at the time of national elections, probably prompting respondents to make this analogy. In this context, many participants saw the ranking grid from the interview a useful tool to structure one’s thoughts about the topic of organ donation. Several respondents took a picture of their ranking grid, to further discuss the topic with their family and to deliberate themselves on the decision whether to register as organ donor.

Based on the present study one could argue that direct questions asking whether people are in favour or against organ donation may have limited information value, especially in relation to understanding decisions to register as organ donor or developing policies to influence them. Attitudes on topics like organ donation are rich and complex that need to be understood before they can be appropriately measured or influenced. As discussed, most respondents appear to have a basic positive attitude to organ donation, but in three of the four viewpoints people holding them experience different barriers that prevent them to translate this positive attitude into action.

Some limitations of this study need to be mentioned. First, we used two theoretical models and adjacent literature for identifying the variety of issues relevant for inclusion in the statement set. Although such a structured approach to the development of the statement set should make it easier to capture the full spectrum of issues, we experienced some difficulties with categorizing statements unambiguously according to these issues. In addition, although complemented with additional issues from the literature, such as ‘moral beliefs’, it may be that certain issues were missed.

We have paid extra attention to potentially missing issues relevant to respondents during the pilot study and the interviews by asking for this specifically, but no missing issues were identified by these respondents. Secondly, during the interview some respondents stated that, when in doubt about the placement of statements according to agreement, they gave higher rankings to statements that they considered more important in the decision to register as organ donor. Hence, in some cases importance was evaluated in addition to agreement as ranking criterion. Although this was not in accordance with the instructions, it is conceivable that respondents would want to give a higher rank to a statement they find important as compared to one they agree with equally but find less important. Because it concerns the relative ranking of very few statements, perhaps one column to the left or the right on the sorting grid, by a small proportion of the participants, we believe this has not affected our findings. Finally, this study identified four prevailing attitudes to organ donation but does not give information about the distribution of these views within the Netherlands. This may be relevant information if one wishes to choose optimal policies to improve donor registration. Identifying the distribution was not the aim of this study and Q-methodology is not the most appropriate method for that purpose, in particular also considering the purposive sampling of respondents. Survey research that matches respondents to these views in a representative sample of the population would be better fit for that purpose, and also verify whether any important viewpoints have been missed [55, 56].

Our findings have some important implications for public policies aimed at promoting deceased organ donation. First, the results indicate that respondents who hold perspective three and four are in a modifiable phase. This could be understood as an opportunity to address existing fears and educate on the organ donation process. Respondents of all views indicated that current media campaigns played little to no role in their decision to register as organ donor because they are focused on the benefits for recipients and do not provide information about aspects of the process relevant to the donor, which suggests an opportunity to develop more informative media campaigns, conveying information for the various viewpoint holders.

Finally, early 2018 a law was passed in the Netherlands that will change donor registration from an opt-in system into an opt-out system effective 2020. All viewpoints show a positive attitude concerning the effects of the opt-out system, indicating that people often forget to register and that the new law will prompt people who do not want to donate their organs to make an active decision to opt out. However, viewpoint two “it does not go with my religion” expresses grave concerns regarding opt-out systems, mainly regarding family members who are not able to access or adequately understand the available information and will stay registered, thereby being considered for organ donation against their religious beliefs. People with the view “my family should decide” will be happy to know that the law provides a voice for the family, although it is not clear how the family will be involved in practice. Finally, it is not immediately clear whether those with the “it’s a good deed, but I‘m doubtful” viewpoint will appreciate the change in default. It may bring forward the fears and unease about the donation process but may also bring relief because the weight of the decision is taken away from them. Given the variety in reactions to this new law that can be expected on the basis of our findings, it seems appropriate to use the two remaining years ahead of the implementation of the law to devise information policies that take away the main concerns of people with reservations towards an opt-out system or organ donation in general, and to promote that people with different views are enabled to enact their preferences under this new system.

## Supporting information

S1 Table Respondent’s answers. (XLSX)
